# A disproportionality analysis of insulin glargine in the overall population and in pregnant women using the FDA adverse event reporting system (FAERS) database

**DOI:** 10.1371/journal.pone.0331489

**Published:** 2025-09-25

**Authors:** Shaozhi Liu, Jun Xu, Zhongwen Yuan, Zhengrong Mei, Shengying Shi, Jinjin Yin, Yanhong Deng

**Affiliations:** Department of Pharmacy, Guangdong Provincial Key Laboratory of Major Obstetric Diseases, Guangdong Provincial Clinical Research Center for Obstetrics and Gynecology, The Third Affiliated Hospital, Guangzhou Medical University, Guangzhou, China; University of British Columbia, CANADA

## Abstract

**Background:**

Insulin glargine (IG) is a commonly prescribed medication for diabetes management in clinical practice, however, there has yet to be a comprehensive systematic study examining its associated adverse events (AEs). In particular, due to the inherent limitations of clinical trials conducted during pregnancy, the safety profile of medications utilized in this period cannot be determined with absolute certainty. This study aims to evaluate the signals of AEs related to IG with in the overall population and among pregnant women, utilizing data from the FDA Adverse Event Reporting System (FAERS) database.

**Methods:**

We employed standardized MedDRA queries to identify adverse event (AE) reports related to pregnancy. Through disproportionate analysis, we identified and analyzed AE reports from the FAERS database spanning January 2004 to June 2024. We used Reporting Odds Ratio (ROR), Proportional Reporting Ratio (PRR), Bayesian Conﬁdence Propagation Neural Network (BCPNN), and Empirical Bayesian Geometric Mean (EBGM) for signal detection. Further identification of signal strength based on the BCPNN method was conducted by categorizing signals into four levels based on the Information Component (IC) value and its 95% confidence interval: weak signals (0 < IC025 ≤ 1.5), moderate signals (1.5 < IC025 ≤ 3), and strong signals (IC025 > 3). Additionally, an analysis of the temporal distribution characteristics of AEs was performed.

**Results:**

We obtained 70 strong or medium signals of AEs for IG in the overall population and 28 positive signals of AEs in pregnant women. In the overall population, the most significant signals included blood glucose abnormal (IC025 = 4.86), blood glucose fluctuation (IC025 = 4.69), blood glucose decreased (IC025 = 4.44), hypoglycaemic seizure (IC025 = 4.44) and hypoglycaemic unconsciousness (IC025 = 4.31). In pregnant women, hypoglycaemia (IC025 = 4.25) was detected as a strong signal, hypoglycaemia neonatal (IC025 = 2.96) as a medium signal, while ketoacidosis (IC025 = 0.76), decreased insulin requirement (IC025 = 0.24), and underweight (IC025 = 0.09) were identified as weak signals. The median time-to-onset of AEs was significantly longer in pregnant women compared to the overall population (186 days vs. 61 days).

**Conclusion:**

This study has identified unexpected AE signals associated with IG in pregnant women. Our research provides valuable evidence for the clinical application of IG, offers real-world data to support safe medication practices during pregnancy, and establishes a foundation for further clinical investigations.

## Introduction

Diabetes has emerged as a significant public health concern globally, and it is linked to an elevated risk of mortality from ischemic heart disease, stroke, chronic liver disease, tumors, and various chronic genitourinary disorders in women [1]. Hyperglycemia in pregnancy includes different types of abnormal glucose metabolism during pregnancy and is increasingly common, affecting an estimated 20 million live births worldwide (about one in six) [2]. It is clearly associated with adverse pregnancy outcomes such as fetal macrosomia, cesarean delivery, premature delivery, preeclampsia, and an increased risk of long-term maternal and fetal metabolic syndrome [3]. Controlling blood sugar levels can significantly reduce the risk of various complications. Insulin glargine (IG) is a crucial medication for the regulation of blood glucose levels and is also utilized to manage elevated blood sugar during pregnancy.

IG is a long-acting insulin analogue created by the addition of two arginine molecules to the carboxyl terminus of the β chain, along with the substitution of aspartic acid with glycine at position 21 of the α chain [4]. Compared to human insulin, IG has a slower onset but a significantly longer duration of action, contributing to its once-daily dosing and flatter pharmacokinetic profile [5], which helps improve postprandial blood glucose control and reduce nighttime hypoglycemia [6]. Large randomized controlled trials, such as ORIGIN and GRACE, have validated the efficacy and safety of IG in the management of diabetes [7,8]. However, most of these trials are conducted under highly controlled, ideal conditions, and the enrolled population is screened and may not fully reflect real-world drug use [9]. Previous research has highlighted several safety concerns associated with IG treatment, such as hypoglycemia and its impact on weight gain [10–12].

A recent review has synthesized evidence from randomized clinical trials and real-world data to evaluate the overall safety profile of IG. [13], however, there is a lack of systematic evaluation of the safety of IG, especially the safety of its use in pregnancy remains unclear. Pregnancy represents a critical period for evaluating drug safety. Physiological alterations during pregnancy, such as enhanced insulin resistance and significant variations in blood glucose levels, can influence the pharmacokinetics and pharmacodynamics of insulin, thereby elevating the risk of adverse reactions [14]. Conversely, insulin can traverse the placental barrier and exert direct or indirect effects on the fetus, potentially leading to adverse pregnancy outcomes including malformations, macrosomia, and neonatal hypoglycemia [15]. IG was previously considered a Class C drug during pregnancy by the U.S. Food and Drug Administration (USFDA), but after June 2015, the product labeling was subsequently updated to include the contraindication: “Not recommended for use during pregnancy based on human data” [16]. While randomized controlled trials specifically investigating IG use in pregnancy are currently lacking, this agent has been extensively utilized in clinical practice for gestational diabetes management [17]. Therefore, assessing the safety of IG during pregnancy is crucial for developing effective blood glucose management strategies and enhancing maternal and neonatal outcomes.

Currently, there is a notable deficiency in systematic and comprehensive studies regarding adverse events (AEs) associated with IG in real-world settings. Real-world data sources, including Medicare databases, electronic medical records, and spontaneous adverse event (AE) reporting systems, offer a more comprehensive and representative assessment of drug safety [18]. Among them, spontaneous reporting serves as the fundamental source of pharmacovigilance information [19]. Therefore, the thorough collection and analysis of these IG-related AEs can provide valuable insights for clinical practice. This study employed the FAERS database to analyze the actual AE signals associated with IG. Additionally, a subgroup analysis of AE signals during pregnancy was conducted to enhance the safety profile of clinical drug use.

## Materials and methods

### Study design and data source

**Fig 1:**
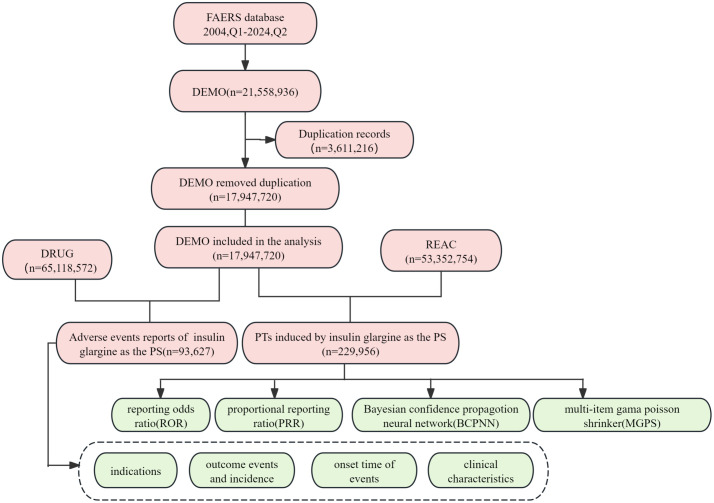
The flow diagram of selecting IG-related AEs in the overall population from FAERS database. Abbreviation: BCPNN, bayesian confidence propagation neural network; EBGM, empirical bayesian geometric mean; MGPS, multi-item gama poisson shrinker; PTs, preferred terms; PS, primary suspect; PRR, proportional reporting ratio; ROR, reporting odds ratio.

**Fig 2:**
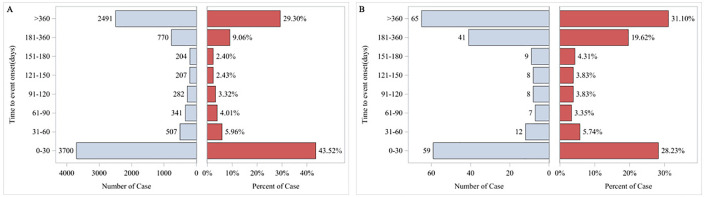
Time to event report distribution of AE reports. (A). Time to event report distribution of AE reports in the overall population. Abbreviation: AE, adverse event. (B). Time to event report distribution of AE reports in pregrant women.

**Fig 3:**
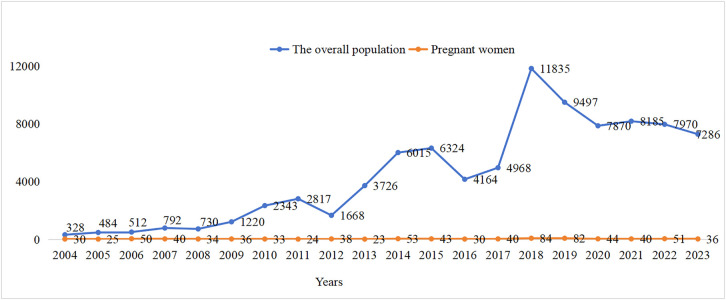
Annual distribution of IG-ralated AE reports.

The study acquired AE data from the FAERS database, a publicly accessible database established in 2004. FAERS is a globally recognized system for reporting AEs and can be downloaded free of charge from its official website. The FAERS database comprises eight distinct types of files: report sources (RPSR), demographic and administrative information (DEMO), drug information (DRUG), indications for use (INDI), start and end dates for reported drugs (THER), adverse event information (REAC), patient outcomes (OUTC), and invalid reports (DELETED) [20]. All documents can be accessed on the FDA website (https://fis.fda.gov/extensions/FPD-QDE-FAERS/FPD-QDE-FAERS.html). This database aggregates reports submitted by healthcare professionals, patients, drug manufacturers, and other stakeholders [21,22]. We obtained AE data pertaining to the use of IG in the overall population from the FAERS database, covering the period from January 2004 to June 2024. This was undertaken to conduct a comprehensive analysis of the AEs associated with IG. A total of 17,947,720 AE reports were recorded in the FAERS database, among which 93,627 reports were linked to IG usage with in the overall population. Concurrently, we extracted 53,352,754 AEs from the FAERS database; out of these, 229,956 were specifically associated with IG used in the overall population (Fig 1). The major induced time of AE with IG in the overall population was shown in Fig 2A. The annual distribution of IG-related AE reports within this demographic was illustrated in Fig 3.

### Identification and retrieval of pregnancy-related reports

**Fig 4:**
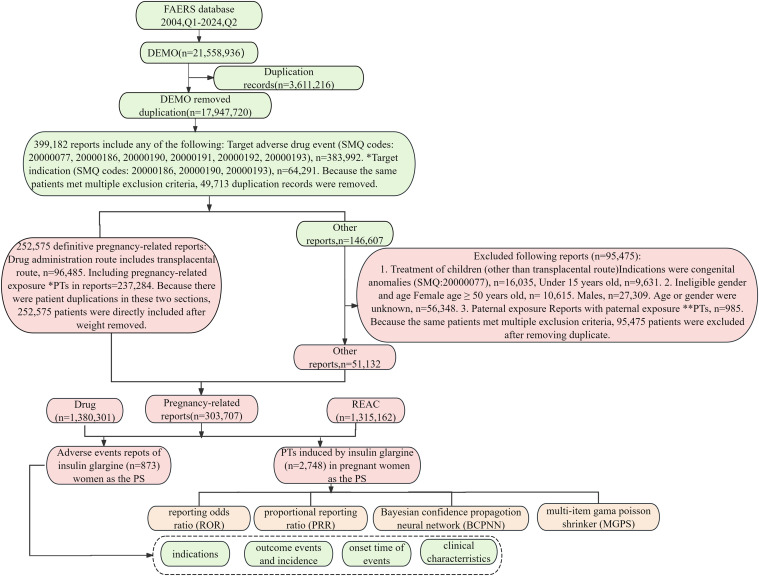
The flow diagram illustrates the procedure for selecting IG-related AEs in pregnant women from the FAERS database. PTs*: Categories include maternal exposure during delivery (10071407), foetal exposure during delivery (10071409), maternal exposure before pregnancy (10071406), maternal exposure during pregnancy (10071408), fetal exposure during pregnancy (10071404), exposure during pregnancy (10073513), maternal exposure timing unspecified (10071415), foetal exposure timing unspecified (10071405), maternal drugs affecting fetus(10026923), drug exposure before pregnancy(10064998). PTs**: Categories include paternal drugs affecting fetus(10050425), exposure via father(10071403), paternal exposure during pregnancy(10,080,091), paternal Exposure timing unspecified(10,080,092), paternal Exposure before Pregnancy (10,080,093) and Maternal Exposure via Partner During Pregnancy(10084938). Abbreviation: BCPNN, bayesian confidence propagation neural network; EBGM, empirical bayesian geometric mean; MGPS, multi-item gama poisson shrinker; PTs, preferred terms; PS, primary suspect; PRR, proportional reporting ratio; ROR, reporting odds ratiop; SMQ, standard MedDRA query.

Since the FAERS database lacks a specific field for identifying reports related to pregnant women, our study employed a standardized MedDRA query (SMQ) in conjunction with a subgroup disproportionality analysis. Additionally, we utilized previously established methods to identify pregnancy-related reports within the FAERS database [19,22]. We collected 17,947,720 records from the FAERS database, as shown in Fig 4. To obtain pregnancy-related reports, we referred to previous research methods [22]. We utilized the SMQ codes 20000077, 20000186, 20000190, 20000191, 20000192, and 20000193 (S1 Table in S1 File), to compile a total of 383,992 records. Additionally, we employed SMQ codes 20000186, 20000190, and 20000193 to extract cases where the indications field included terms related to pregnant mothers, resulting in a total of 64,291 records. To account for potential duplicates among patients, we ultimately included a total of 399,182 unique patients after removing duplicate entries.

Cases involving pregnancy-related preferred terms (PTs) or transplacental administration were classified as clearly pregnancy-related reports. After the removal of duplicates, a total of 252,575 pregnancy-related records were obtained. Additionally, 95,475 reports were excluded due to gender incompatibility, age factors, paternal exposure and so on. Following the elimination of duplicates from the remaining data set, we identified an additional 51,132 records. In all, our analysis identified 303,707 pregnancy-related AE reports in the database, among which 873 cases involved IG exposure during gestation. We extracted 1,315,162 pregnancy-related AEs, among which 2,748 were linked to IG usage during gestation as illustrated in Fig 4. The annual distribution of AE reports related to IG used during pregnancy was presented in Fig 3, the major onset timing is depicted in Fig 2B.

### Analysis of signal strength of AEs

In our study, we employed disproportionality analysis, a widely recognized method, to identify potential signals linking IG with AEs. This approach assesses the correlation between the medication and AEs by examining the observed frequencies in both exposed and non-exposed populations, utilizing a 2 × 2 contingency table as depicted in S2 Table in S1 File. We concurrently applied four methods to detect signals of adverse drug events: the Reporting Odds Ratio (ROR), the Proportional Reporting Ratio (PRR), the Bayesian Confidence Propagation Neural Network (BCPNN), and the Empirical Bayesian Geometric Mean (EBGM). For the statistical analysis conducted with SAS9.4, the thresholds for these methods were set as follows: a ≥ 3, a lower limit of the ROR 95% confidence interval greater than 1, PRR ≥ 2, chi-square ≥ 4, IC-2SD > 0, and EBHM05 > 2 [22]. In our study, the AEs identified as positive signals were required to fulfill the criteria established by the four aforementioned methods. This indicates a potential correlation between the drug and the event. In order to further reveal the signal strength, we further classified according to the BCPNN method. In the BCPNN method, an IC025 value between 0 and 1.5 is categorised as a weak signal, a value between 1.5 and 3 is considered a medium signal, and a value greater than 3 is regarded as a strong signal [23]. Additionally, the time to onset (TTO) was defined as the interval between the initiation of IG and the occurrence of AEs. Prior to analysis, we conducted a deduplication process and eliminated any invalid data. In this study, we utilized median values (interquartile range [IQR]) along with minimum and maximum values to assess the characteristics of TTO [24]. All data processing and statisticalanalyses were performed used SAS 9.4 (SAS Institute Inc., Cary, NC, United States) [20].

## Results

### Basic information about AEs of IG

The basic characteristics of IG were shown in Table 1. For IG in the overall population, female accounted for 52.24% of the reports, and the majority of AE reports came from people aged 45–64 years, although a significant number were unknown age. Most of the reporters were consumers (81.83%), and the main reporting country was North America (86.57%). The major outcome was other (27.12%). The median time for AE to occur with IG was 61.00 (1.00,424.00) days.

**Table 1:** Characteristics of AEs reports associated with IG.

Characteristics	IG used in pregnancy, n (%)	IG used in the overall population, n (%)
**Sex**		
Female (%)	695 (79.61)	48909 (52.24)
Male (%)	97 (11.11)	36166 (38.63)
Not Specified (%)	81 (9.28)	8552 (9.13)
**Age(yaers)**		
< 18(%)	74 (8.47)	977 (1.04)
18-44 (%)	383 (43.87)	3978 (4.25)
45-64 (%)	15 (1.72)	20595 (22.00)
65-74 (%)	NA	17550 (18.74)
≥ 75 (%)	NA	13867 (14.81)
NotSpecified (%)	401 (45.93)	36660 (39.16)
**Reporter**		
Consumer (%)	433 (49.60)	76618 (81.83)
Not Specified (%)	80 (9.16)	20 (0.02)
Other health-professional (%)	88 (10.08)	1680 (1.79)
Pharmacist (%)	41 (4.70)	2889 (3.09)
Physician (%)	231 (26.46)	8233 (8.79)
**Geographical distribution**		
North America (%)	301 (34.48)	81056 (86.57)
Europe (%)	250 (28.64)	4070 (4.35)
South America (%)	111 (12.71)	2965 (3.17)
Asia (%)	81 (9.28)	2822 (3.01)
Africa (%)	60 (6.87)	1494 (1.60)
Oceania (%)	7 (0.80)	482 (0.51)
Missing (%)	53 (6.07)	690 (0.74)
Others (%)	10 (1.15)	48 (0.05)
**Outcomes**		
Life-Threatening (%)	24 (2.75)	1071 (1.14)
Hospitalization-Initial or Prolonged (%)	227 (26.00)	13690 (14.62)
Disability (%)	7 (0.80)	1789 (1.91)
Death (%)	2 6(2.98)	3104 (3.32)
Congenital Anomaly (%)	103 (11.80)	114 (0.12)
Required Intervention to Prevent Permanent Impairment/Damage (%)	0 (0.00)	95 (0.10)
** **Other (%)	431 (49.37)	25387 (27.12)
**Adverse event occurrence time(days)**		
0-30d (%)	59 (6.76)	3700 (3.95)
31-60d (%)	12 (1.37)	507 (0.54)
61-90d (%)	7 (0.80)	341 (0.36)
91-120d (%)	8 (0.92)	282 (0.30)
121-150d (%)	8 (0.92)	207 (0.22)
151-180d (%)	9(1.03)	204 (0.22)
181-360d (%)	41 (4.70)	770 (0.82)
> 360d (%)	65 (7.45)	2491 (2.66)
Missing or outlier (less than 0) (%)	664 (76.06)	85125 (90.92)
**Adverse event occurrence time(days)**		
N (Missing)	209 (664)	8502 (85125)
Mean (SD)	548.68 (1079.65)	452.85 (938.67)
Median (Q1,Q3)	186.00 (21.00,476.00)	61.00 (1.00,424.00)
Min,Max	0.00,7595.00	0.00,14340.0

For IG in pregnant women, most of the reported ages were notspecified (45.93%). Most of the reporters were consumer (49.60%), and the majority of reports were from North America (34.48%). Serious outcomes of IG used during pregnancy mainly included other (49.37%). The median time to AE occurrence was 186.00 (21.00,476.00) days.

### Signals detection associated with IG

#### Signals detection at SOCs levels.

**Fig 5:**
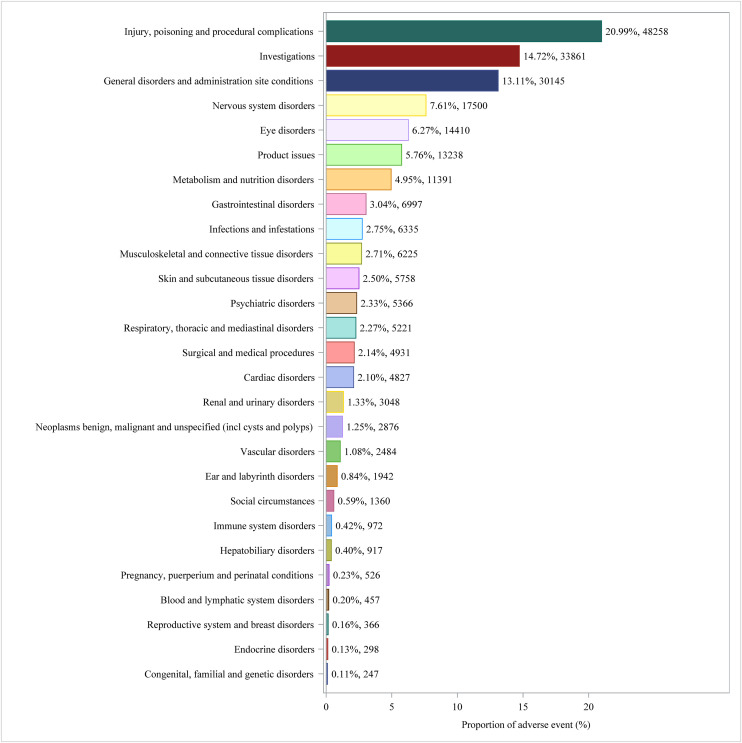
Proportion of AEs by SOCs in the overall population.

SOC analysis of IG-associated AEs in the overall population (S3 Table in S1 File) identified 27 affected organ systems, with proportional distribution visualized in Fig 5. At the SOC level, IG-associated AEs were most frequently observed in: injury, poisoning and procedural complications (20.99%), investigations (14.72%), and general disorders and administration sites conditions (13.11%). Significant adverse signals reported in SOC were product issues (n = 13,238, ROR 3.80, PRR 3.64, IC 1.85, EBGM 3.60), eye disorders (n = 14,410, ROR 3.34, PRR 3.19, ROR 3.34, IC 1.66, EBGM 3.16) and investigations (n = 33,861, ROR 2.65, PRR 2.40, IC 1.26, EBGM 2.39).

**Fig 6:**
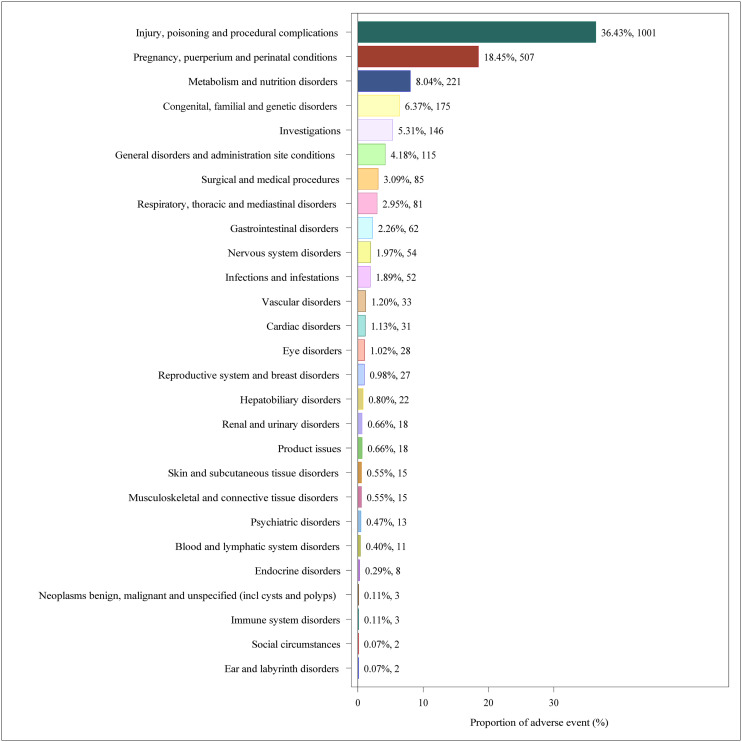
Proportion of AEs by SOCs in pregnant women.

AE signals at the SOC of IG in pregnant women, shown in S4 Table in S1 File, showed that 27 SOC were involved in adverse reactions, and the distribution ratio was shown in Fig 6. By SOC level, the most frequently reported AEs were: injury, poisoning and procedural complications (36.43%), pregnancy, puerperium and perinatal conditions (18.45%) and metabolism and nutrition disorders (8.04%). The significant adverse signals reported in SOC were metabolism and nutrition disorders (n = 221, ROR 6.80, PRR 6.33, IC 2.65, EBGM 6.26), injury, poisoning and procedural complications (n = 1,001, ROR 1.79, PRR 1.50, IC 0.59, EBGM 1.50) and Endocrine disorders (n = 8, ROR 1.75, PRR 1.74, IC 0.80, EBGM 1.74).

#### Signals detection at PTs levels.

In the BCPNN method, based on the IC025 values, a total of 70 AEs were identified as signals associated with IG used in the overall population, including 18 strong signals (IC025 > 3) and 52 medium signals (1.5 < IC025 ≤ 3). The strongest signals were mainly from the SOCs of Metabolism and nutrition disorders, Investigations, Surgical and medical procedures, Eye disorders, and Nervous system disorders. The top five strongest signals included blood glucose abnormal (IC025 = 4.86), blood glucose fluctuation (IC025 = 4.69), blood glucose decreased (IC025 = 4.44), hypoglycaemic seizure (IC025 = 4.44) and hypoglycaemic unconsciousness (IC025 = 4.31). In the SOC of Metabolism and nutrition disorders, 9 AEs were identified as signals, including 5 strong signals and 4 medium signals. The strong signals were hypoglycaemia (IC025 = 4.26), shock hypoglycaemic (IC025 = 3.68), hypoglycaemia unawareness (IC025 = 3.30), insulin resistance (IC025 = 3.02) and dawn phenomenon (IC025 = 3.01). For the Investigations SOC, 9 signals were detected, consisting of 5 strong and 4 medium signals. The strong signals included blood glucose abnormal (IC025 = 4.86), blood glucose fluctuation (IC025 = 4.69), blood glucose decreased (IC025 = 4.44), anti-insulin antibody positive (IC025 = 3.49), and glycosylated haemoglobin decreased (IC025 = 3.44). In addition, we also identified some strong signals related to eye disorders, as well as other moderate-intensity signals including pancreatic disorder, frustration tolerance decreased, hypoacusis, and neuropathic arthropathy, shown in Table 2.

**Table 2:** Signal strength of AEs at the Preferred Term(PT) level ranked by IC025 in the overall population.

SOC/PT	Case reports	ROR (95% CI)	PRR (95% CI)	Chi Square	IC (IC025)	EBGM (EBGM05)
**Metabolism and nutrition disorders**						
Hypoglycaemia	3689	22.04 (21.31,22.81)	21.71 (20.99,22.45)	66661.2	4.32 (4.26)	19.93 (19.26)
Shock hypoglycaemic	49	29.03 (21.57,39.07)	29.02 (21.57,39.06)	1177.91	4.69 (3.68)	25.90 (19.24)
Hypoglycaemia unawareness	34	25.34 (17.78,36.11)	25.34 (17.78,36.10)	716.25	4.52 (3.30)	22.93 (16.09)
Insulin resistance	86	11.85 (9.54,14.72)	11.85 (9.54,14.71)	812.45	3.50 (3.02)	11.32 (9.11)
Ketoacidosis	266	9.75 (8.63,11.03)	9.74 (8.62,11.01)	2002.37	3.23 (3.01)	9.39 (8.30)
Dawn phenomenon	17	51.68 (30.54,87.44)	51.67 (30.54,87.43)	690.36	5.41 (2.94)	42.41 (25.07)
Hyperinsulinaemic hypoglycaemia	16	25.15 (15.01,42.13)	25.14 (15.01,42.12)	334.53	4.51 (2.58)	22.77 (13.59)
Ketosis	30	9.46 (6.56,13.62)	9.45 (6.56,13.62)	217.90	3.19 (2.32)	9.12 (6.33)
Hypoglycaemia neonatal	40	6.56 (4.79,8.99)	6.56 (4.79,8.99)	183.40	2.68 (2.04)	6.41 (4.68)
**Investigations**						
Blood glucose abnormal	2165	35.77 (34.18,37.43)	35.44 (33.88,37.08)	62846.1	4.95 (4.86)	30.86 (29.49)
Blood glucose fluctuation	1188	31.88 (30.00,33.87)	31.72 (29.85,33.69)	31080.1	4.81 (4.69)	28.01 (26.36)
Blood glucose decreased	4213	25.31 (24.51,26.14)	24.87 (24.10,25.66)	87201.2	4.49 (4.44)	22.55 (21.84)
Anti-insulin antibody positive	30	40.30 (27.34,59.39)	40.29 (27.34,59.38)	978.81	5.11 (3.49)	34.46 (23.38)
Glycosylated haemoglobin decreased	88	16.88 (13.59,20.96)	16.87 (13.59,20.95)	1224.45	3.98 (3.44)	15.79 (12.72)
Blood ketone body	26	21.15 (14.16,31.61)	21.15 (14.15,31.60)	457.25	4.28 (2.95)	19.46 (13.02)
Blood ketone body increased	26	10.06 (6.79,14.90)	10.06 (6.79,14.90)	203.31	3.28 (2.30)	9.68 (6.54)
Blood insulin increased	20	10.60 (6.77,16.59)	10.60 (6.77,16.59)	166.20	3.35 (2.18)	10.18 (6.50)
Blood ketone body present	10	15.30 (8.07,29.02)	15.30 (8.07,29.01)	125.34	3.85 (1.80)	14.41 (7.60)
**General disorders and administration site conditions**						
Injection site injury	150	9.36 (7.95,11.02)	9.35 (7.94,11.01)	1075.14	3.17 (2.86)	9.03 (7.67)
Injection site hypertrophy	22	22.59 (14.58,35.00)	22.59 (14.58,35.00)	413.48	4.37 (2.85)	20.67 (13.34)
Injection site atrophy	43	6.85 (5.06,9.27)	6.85 (5.06,9.27)	208.49	2.74 (2.12)	6.68 (4.93)
Injection site scar	59	5.88 (4.54,7.62)	5.88 (4.54,7.62)	233.17	2.53 (2.04)	5.76 (4.45)
Injection site haemorrhage	1236	4.36 (4.12,4.61)	4.34 (4.10,4.59)	3121.83	2.10 (2.01)	4.28 (4.04)
Injection site discolouration	179	3.99 (3.44,4.62)	3.98 (3.44,4.62)	393.38	1.98 (1.74)	3.93 (3.39)
Injection site bruising	976	3.50 (3.29,3.73)	3.49 (3.28,3.72)	1710.77	1.79 (1.69)	3.45 (3.24)
Injection site pain	3496	3.35 (3.24,3.47)	3.32 (3.21,3.43)	5606.14	1.72 (1.67)	3.28 (3.18)
Hunger	154	3.76 (3.20,4.41)	3.76 (3.20,4.40)	306.43	1.89 (1.63)	3.71 (3.16)
**Surgical and medical procedures**						
Cataract operation	244	11.78 (10.36,13.40)	11.77 (10.35,13.39)	2288.82	3.49 (3.24)	11.25 (9.89)
Eye operation	152	10.65 (9.05,12.53)	10.64 (9.04,12.52)	1269.28	3.35 (3.03)	10.22 (8.68)
Cardiac operation	249	8.44 (7.43,9.57)	8.43 (7.43,9.56)	1572.98	3.03 (2.80)	8.17 (7.20)
Eye laser surgery	28	14.19 (9.69,20.78)	14.19 (9.69,20.78)	323.31	3.75 (2.68)	13.42 (9.16)
Vascular graft	99	8.46 (6.93,10.34)	8.46 (6.92,10.34)	628.37	3.04 (2.64)	8.20 (6.71)
Coronary artery bypass	107	4.57 (3.78,5.54)	4.57 (3.78,5.54)	292.80	2.17 (1.84)	4.50 (3.72)
Retinal operation	11	12.40 (6.76,22.74)	12.40 (6.76,22.74)	109.37	3.56 (1.78)	11.82 (6.44)
Laser therapy	11	12.40 (6.76,22.74)	12.40 (6.76,22.74)	109.37	3.56 (1.78)	11.82 (6.44)
**Eye disorders**						
Visual impairment	5698	13.04 (12.69,13.40)	12.74 (12.41,13.08)	58561.2	3.60 (3.56)	12.13 (11.81)
Retinopathy	182	13.29 (11.44,15.43)	13.28 (11.43,15.41)	1953.75	3.66 (3.35)	12.61 (10.86)
Macular degeneration	369	9.08 (8.18,10.07)	9.06 (8.17,10.06)	2547.73	3.13 (2.95)	8.76 (7.89)
Cataract	1525	7.37 (7.00,7.76)	7.33 (6.96,7.71)	8083.34	2.83 (2.75)	7.13 (6.78)
Eye haemorrhage	380	7.63 (6.89,8.46)	7.62 (6.88,8.44)	2117.16	2.89 (2.72)	7.41 (6.69)
Blindness	1004	6.95 (6.53,7.40)	6.92 (6.50,7.37)	4943.15	2.76 (2.65)	6.75 (6.34)
Retinal disorder	69	7.15 (5.62,9.08)	7.14 (5.62,9.08)	353.72	2.80 (2.33)	6.96 (5.48)
Retinopathy proliferative	10	13.92 (7.35,26.35)	13.92 (7.35,26.34)	113.07	3.72 (1.74)	13.18 (6.96)
Blindness unilateral	323	6.31 (5.65,7.05)	6.30 (5.64,7.04)	1403.06	2.62 (2.44)	6.16 (5.52)
Eye disorder	740	6.29 (5.84,6.76)	6.27 (5.83,6.74)	3192.20	2.62 (2.50)	6.13 (5.70)
Visual acuity reduced	809	6.10 (5.69,6.54)	6.08 (5.67,6.52)	3348.06	2.57 (2.46)	5.95 (5.55)
Glaucoma	390	5.52 (4.99,6.10)	5.51 (4.99,6.09)	1407.58	2.43 (2.27)	5.41 (4.89)
Retinal haemorrhage	115	4.37 (3.64,5.26)	4.37 (3.63,5.26)	293.45	2.11 (1.80)	4.31 (3.58)
**Social circumstances**						
Corrective lens user	45	20.47 (15.09,27.76)	20.46 (15.09,27.76)	765.28	4.24 (3.32)	18.88 (13.92)
Disability	533	7.52 (6.90,8.20)	7.50 (6.88,8.18)	2910.96	2.87 (2.72)	7.30 (6.70)
Wheelchair user	120	7.45 (6.21,8.94)	7.45 (6.21,8.93)	649.00	2.86 (2.52)	7.25 (6.04)
Hearing disability	16	11.41 (6.91,18.85)	11.41 (6.91,18.85)	144.78	3.45 (2.07)	10.92 (6.61)
Hearing aid user	12	10.01 (5.62,17.84)	10.01 (5.62,17.84)	93.25	3.27 (1.71)	9.63 (5.41)
**Nervous system disorders**						
Hypoglycaemic seizure	117	38.25 (31.45,46.52)	38.23 (31.44,46.49)	3639.69	5.04 (4.41)	32.94 (27.09)
Hypoglycaemic unconsciousness	208	28.82 (24.95,33.28)	28.79 (24.93,33.25)	4961.44	4.68 (4.31)	25.71 (22.26)
Hypoglycaemic coma	233	22.90 (20.01,26.20)	22.88 (20.00,26.17)	4435.22	4.39 (4.07)	20.90 (18.27)
Hypoglycaemic encephalopathy	26	19.07 (12.78,28.45)	19.07 (12.78,28.44)	411.19	4.14 (2.87)	17.69 (11.86)
Dementia Alzheimer’s type	188	5.44 (4.71,6.29)	5.44 (4.71,6.29)	665.89	2.42 (2.17)	5.34 (4.62)
Dementia	439	4.50 (4.09,4.95)	4.49 (4.09,4.94)	1170.35	2.15 (2.00)	4.43 (4.03)
Dyslexia	25	6.03 (4.05,8.97)	6.03 (4.05,8.97)	102.19	2.56 (1.74)	5.90 (3.97)
Cerebrovascular accident	2003	3.08 (2.95,3.22)	3.06 (2.93,3.20)	2754.47	1.60 (1.54)	3.04 (2.90)
**Skin and subcutaneous tissue disorders**						
Lipohypertrophy	31	13.62 (9.48,19.56)	13.61 (9.48,19.56)	342.17	3.69 (2.71)	12.91 (8.99)
Cutaneous amyloidosis	12	45.45 (24.47,84.40)	45.45 (24.47,84.39)	435.86	5.25 (2.43)	38.14 (20.54)
Lipodystrophy acquired	38	4.76 (3.45,6.56)	4.76 (3.45,6.56)	110.61	2.23 (1.63)	4.68 (3.40)
**Injury, poisoning and procedural complications**						
Lack of injection site rotation	49	9.36 (7.03,12.45)	9.36 (7.03,12.45)	351.41	3.17 (2.54)	9.03 (6.79)
Exposure via skin contact	169	6.31 (5.42,7.35)	6.31 (5.41,7.35)	734.89	2.62 (2.36)	6.17 (5.29)
**Other SOC**						
Pancreatic disorder	134	7.91 (6.66,9.40)	7.91 (6.66,9.39)	782.03	2.94 (2.62)	7.68 (6.47)
Frustration tolerance decreased	151	3.45 (2.94,4.06)	3.45 (2.94,4.05)	259.23	1.77 (1.51)	3.42 (2.91)
Hypoacusis	1393	7.88 (7.47,8.31)	7.83 (7.43,8.26)	8038.71	2.93 (2.84)	7.61 (7.21)
Neuropathic arthropathy	24	9.69 (6.44,14.58)	9.69 (6.44,14.58)	179.57	3.22 (2.22)	9.34 (6.21)

Note1:ranked by IC025.

Note2:Signals are detected when all the following criteria are met:a ≥ 3, PRR ≥ 2 and Chi-Square ≥ 4, lower limit of 95% CI of ROR > 1, IC025 ≥ 1.5, EBGM05 > 2.

In pregnant women, we present AE signals of all levels, including strong, medium, and weak signals, while in the overall population analysis, we only report AEs with medium and strong signals. This difference is due to the fact that pregnancy is a special physiological period, and the response of the mother and fetus to medications may differ from that of the general population. Even relatively weak AE signals may have significant impacts on the mother and fetus, making it necessary to comprehensively evaluate and report them. Based on the IC025 values, a total of 28 AEs were identified as signals associated with IG use in the overall population, including 1 strong signals (IC025 > 3), 4 medium signals (1.5 < IC025 ≤ 3) and 23 weak signals (IC025 < 1.5). In the Metabolism and nutrition disorders SOC, hypoglycaemia (IC025 = 4.25) was detected as a strong signal, hypoglycaemia neonatal (IC025 = 2.96) as a medium signal, while ketoacidosis (IC025 = 0.76), decreased insulin requirement (IC025 = 0.24), and underweight (IC025 = 0.09) were identified as weak signals. The Investigations SOC contained medium signals for blood glucose decreased (IC025 = 2.16), blood glucose fluctuation (IC025 = 2.07) and blood glucose abnormal (IC025 = 1.57). The Pregnancy, puerperium and perinatal conditions SOC included several weak signals such as jaundice neonatal (IC025 = 1.29), foetal distress syndrome (IC025 = 1.08), abortion (IC025 = 1.06), foetal disorder (IC025 = 0.84), polyhydramnios (IC025 = 0.74), foetal hypokinesia (IC025 = 0.71), foetal macrosomia (IC025 = 0.49), and abnormal labour (IC025 = 0.23). Other weak signals were distributed across various SOCs, included hypoglycaemic seizure (IC025 = 0.76), intensive care (IC025 = 0.81), hospitalisation (IC025 = 0.99), kidney malformation (IC025 = 0.69), congenital bladder anomaly (IC025 = 0.19), gastrointestinal malformation (IC025 = 0.14) and so on, shown in Table 3.

**Table 3:** Signal strength of AEs at the PT level ranked by IC025 in pregnant women.

System Organ Class(SOC)/Preferred Term(PT)	Case reports	ROR (95% CI)	PRR (95% CI)	Chi Square	IC (IC025)	EBGM (EBGM05)
**Metabolism and nutrition disorders**						
Hypoglycaemia	73	39.42 (30.96,50.18)	38.40 (30.34,48.59)	2462.82	5.15 (4.25)	35.61 (27.97)
Hypoglycaemia neonatal	40	14.69 (10.70,20.17)	14.49 (10.61,19.81)	488.24	3.82 (2.96)	14.10 (10.27)
Ketoacidosis	4	24.53 (8.97,67.04)	24.49 (8.97,66.85)	85.74	4.55 (0.76)	23.35 (8.54)
Decreased insulin requirement	3	286.87 (68.52,1200.97)	286.55 (68.51,1198.49)	533.55	7.49 (0.24)	179.47 (42.87)
Underweight	3	8.49 (2.71,26.59)	8.48 (2.71,26.53)	19.44	3.06 (0.09)	8.35 (2.66)
**Nervous system disorders**						
Hypoglycaemic seizure	4	239.14 (71.97,794.64)	238.79 (71.95,792.56)	631.47	7.32 (0.76)	159.53 (48.01)
**Investigations**						
Blood glucose decreased	13	16.67 (9.58,29.02)	16.60 (9.56,28.82)	184.24	4.01 (2.16)	16.08 (9.24)
Blood glucose fluctuation	9	43.56 (21.99,86.27)	43.42 (21.97,85.82)	341.91	5.32 (2.07)	39.88 (20.14)
Blood glucose abnormal	7	24.46 (11.44,52.33)	24.40 (11.43,52.10)	149.48	4.54 (1.57)	23.26 (10.88)
**Injury, poisoning and procedural complications**						
Exposure during pregnancy	518	3.38 (3.07,3.72)	2.93 (2.71,3.17)	700.44	1.55 (1.40)	2.92 (2.65)
**Surgical and medical procedures**						
Hospitalisation	8	6.22 (3.09,12.50)	6.20 (3.09,12.45)	34.48	2.62 (0.99)	6.14 (3.05)
Intensive care	4	34.16 (12.38,94.27)	34.11 (12.38,94.01)	120.01	5.00 (0.81)	31.91 (11.56)
**Congenital, familial and genetic disorders**						
Kidney malformation	5	8.05 (3.32,19.51)	8.04 (3.32,19.44)	30.32	2.99 (0.69)	7.92 (3.27)
Congenital bladder anomaly	3	11.47 (3.65,36.08)	11.46 (3.65,36.00)	27.98	3.49 (0.19)	11.22 (3.57)
Gastrointestinal malformation	3	9.63 (3.07,30.20)	9.62 (3.07,30.14)	22.71	3.24 (0.14)	9.45 (3.01)
**Pregnancy, puerperium and perinatal conditions**						
Jaundice neonatal	16	4.98 (3.04,8.16)	4.96 (3.03,8.10)	50.06	2.30 (1.29)	4.91 (3.00)
Foetal distress syndrome	16	4.11 (2.51,6.74)	4.10 (2.51,6.69)	37.16	2.02 (1.08)	4.07 (2.48)
Abortion	18	3.84 (2.41,6.11)	3.82 (2.41,6.07)	37.25	1.93 (1.06)	3.80 (2.39)
Foetal disorder	8	5.17 (2.57,10.38)	5.16 (2.57,10.34)	26.53	2.35 (0.84)	5.11 (2.54)
Polyhydramnios	8	4.60 (2.29,9.23)	4.59 (2.29,9.19)	22.24	2.19 (0.74)	4.55 (2.27)
Foetal hypokinesia	7	5.09 (2.42,10.73)	5.08 (2.41,10.69)	22.71	2.33 (0.71)	5.04 (2.39)
Foetal macrosomia	4	9.42 (3.50,25.37)	9.41 (3.50,25.30)	29.49	3.21 (0.49)	9.25 (3.44)
Abnormal labour	3	13.40 (4.25,42.25)	13.39 (4.25,42.15)	33.46	3.71 (0.23)	13.05 (4.14)
**Infections and infestations**						
Kidney infection	3	9.08 (2.89,28.46)	9.07 (2.90,28.40)	21.14	3.16 (0.12)	8.92 (2.84)
**General disorders and administration site conditions**						
Injection site pain	17	3.70 (2.29,5.98)	3.69 (2.29,5.93)	33.07	1.87 (0.99)	3.66 (2.27)
Injection site haemorrhage	3	8.20 (2.62,25.67)	8.19 (2.62,25.62)	18.61	3.01 (0.08)	8.07 (2.57)
**Respiratory, thoracic and mediastinal disorders**						
Respiratory arrest	3	8.15 (2.60,25.53)	8.14 (2.60,25.47)	18.48	3.00 (0.08)	8.02 (2.56)
**Hepatobiliary disorders**						
Jaundice	10	5.82 (3.12,10.87)	5.80 (3.11,10.81)	39.30	2.52 (1.13)	5.75 (3.08)

Note1:ranked by IC025.

Note2:Signals are detected when all the following criteria are met:a ≥ 3, PRR ≥ 2 and Chi-Square ≥ 4, lower limit of 95% CI of ROR > 1, IC025 > 0, EBGM05 > 2.

#### TTO analysis of IG-related AEs.

**Fig 7:**
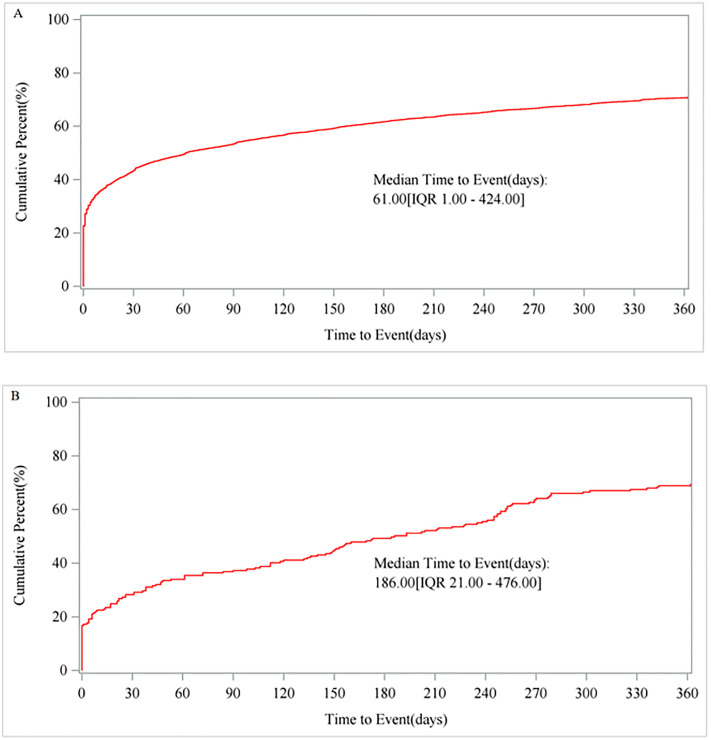
Cumulative incidence of AEs. (A). Cumulative incidence of AEs in the overall population. (B). Cumulative incidence of AEs in pregnant women.

The TTO of AEs related to IG, shown in Fig 7. In the overall population (Fig 7A), the cumulative incidence of AEs steadily increases over time, indicated that long-term use of glycerol insulin might increase the risk of AEs, with a median TTO of 61 days. In contrast to the overall population, pregnant women exhibited a significantly accelerated cumulative incidence of AEs (Fig 7B), with a median TTO of 186 days, suggested pregnancy might potentiate the risks associated with IG therapy.

In the overall population, hypoglycemia was the most common AE in terms of metabolic and nutritional disorders, with 3,689 cases and a median TTO of 131 days. Other common AEs included ketoacidosis, insulin resistance, and neonatal hypoglycemia. Among various test abnormalities, the most reports were for blood glucose decreased (4,213 cases) and blood glucose abnormal (2,165 cases), with median TTO of 22 days and 15 days, respectively. In terms of general disorders and administration site conditions, injection site pain (3,496 cases) and Injection site haemorrhage (1,236 cases) were the most common, with median TTO of 15 days and 28 days, shown in S5 Table in S1 File.

In pregnant women, metabolic and nutritional disorders remained the main issue. The highest number of cases involved fetal exposure during pregnancy, totaling 518 cases with a median TTO of 47 days. This was followed by reports of hypoglycemia (73 cases) and hypoglycaemia neonatal (40 cases), with median TTO of 1,128 days and 220 days, shown in S6 Table in S1 File.

## Discussion

We conducted a systematic and comprehensive analysis of AEs associated with IG, as reported in the FAERS database from January 2004 to June 2024. Additionally, we performed a subgroup analysis focusing on AEs related to the use of IG during pregnancy. For the overall population, significant AEs at SOC levels included product issues, eye disorders and investigations. For pregnant women, significant AEs at SOC levels included injury, poisoning and procedural complications, pregnancy, puerperium and perinatal conditions and metabolism and nutrition disorders. The specific spectrum of AEs differs between the two population groups.

Among the overall population, the primary types of adverse reactions include issues related to product issues, eye disorders, investigations and metabolism and nutrition disorders. In pregnant women, the prevalence of metabolism and nutrition disorders is notably higher. This suggests that when evaluating and managing the safety of insulin, It is crucial to take into account factors that are specific to different populations. Further analysis of signal strength revealed that hypoglycemia-related events were the most significant signal across both populations, highlighting hypoglycemia as the primary safety concern associated with insulin therapy. This finding aligns with the outcomes of previous research, where hypoglycemic events have emerged as a top priority in the safety monitoring and management of insulin medications [25,26]. Severe hypoglycemia can lead to cognitive impairment, cardiovascular events, falls, traffic accidents and other serious consequences, even life-threatening [27–29]. Consequently, it is imperative to enhance hypoglycemia risk education for patients to bolster their self-management and coping skills. Concurrently, healthcare providers must tailor insulin dosages and refine administration schedules based on the unique characteristics of each patient, such as age, renal function, and dietary preferences, to mitigate the risk of hypoglycemic episodes [30].

Beyond hypoglycemia, this study also identified visual impairment and retinopathy as significant signals within eye disorders, indicating that eye complications could be an additional safety concern associated with prolonged insulin use. Epidemiological research has demonstrated that diabetic retinopathy is a leading cause of blindness among adults [31]. Long-term hyperglycemia can cause retinal microvascular damage, resulting in exudation, bleeding, ischemia and other pathological changes, and eventually impair visual function [32]. Insulin therapy may accelerate this process, particularly in situations characterized by significant glycemic fluctuations and frequent episodes of hypoglycemia [33]. Consequently, for individuals on long-term insulin therapy, routine ocular examinations and prompt interventions are crucial. Upon detection of retinopathy, timely referral to an ophthalmologist is imperative to initiate treatments such as laser photocoagulation and vitrectomy. These measures are vital in slowing the progression of the disease and preserving visual acuity [34].

Moderate-intensity signals were also observed for insulin-specific administration site reactions, including injection-site injury and hypertrophy. These issues may be attributed to factors such as non-standard insulin injection techniques, site fixation, and the reuse of needles [35]. Injection site reactions not only affect drug absorption and efficacy, but may also lead to decreased patient compliance and treatment failure [36]. Therefore, enhancing patient education on proper insulin injection techniques, implementing regular rotation of injection sites, and utilizing thin, short insulin pen needles can effectively mitigate the risk of injection site complications [37]. For injection sites exhibiting induration, hypertrophy, or other alterations, it is advised to temporarily suspend injections and resume only after the local reactions have subsided. If required, a dermatology consultation should be sought to establish a diagnosis and formulate an appropriate management plan.

For pregnant women, the signal strength analysis of AEs associated with IG indicated that hypoglycemia, fetal abnormalities, and pregnancy complications were significant safety concerns [38]. Consequently, achieving optimal blood sugar management and maintaining glucose levels within a safe range during pregnancy are essential for enhancing both maternal and neonatal outcomes. However, the physiological changes that occur during pregnancy, including increased insulin resistance, significant blood sugar fluctuations, and a heightened risk of hypoglycemia, present considerable challenges to effective blood sugar control [39]. Thus, it is paramount for safeguarding the health of both mother and child to devise a personalized blood glucose monitoring regimen, to strike a balance between the risks of maternal hypoglycemia and fetal exposure to hyperglycemia, and to fine-tune insulin dosages to accommodate the evolving demands of pregnancy [40].

In addition, in our study, IG-related teratogenic signals were also found, and the results showed weak signals, including kidney malformation (n = 5), congenital bladder anomaly (n = 3) and gastrointestinal malformation (n = 3). Although these teratogenic signals have not yet established a definitive causal link, they nonetheless sound a clear note of caution for clinicians prescribing insulin in pregnancy. With the discontinuation of insulin detemir (previously the only FDA-approved insulin for pregnancy), IG and insulin degludec have emerged as the recommended alternative therapies for gestational diabetes management. Robust, multicenter randomized controlled trials are now urgently required to delineate any dose-response relationship, identify critical windows of exposure, and clarify the role of genetic susceptibility in mediating fetal structural malformations. Such studies should deploy standardized fetal-outcome assessments and long-term follow-up to furnish the high-grade evidence needed for precision prescribing and informed risk management. Prenatal screening and diagnostic procedures are crucial for the early detection and management of congenital anomalies. Routine screening measures include first-trimester Down syndrome serological screening, mid-trimester maternal serum alpha-fetoprotein (AFP) testing, and systematic ultrasound examinations [41].

The timing of AEs associated with IG was also subjected to analysis. The findings revealed that, across the overall population, the cumulative incidence of AEs rose progressively with time, with a median onset at 61 days. This indicates that the prolonged use of insulin may be associated with an increased risk of AEs. This association may stem from factors such as drug accumulation, the development of drug resistance, and the emergence of complications due to the extended duration of insulin therapy [42]. Consequently, clinicians ought to adjust the insulin dosage dynamically in response to fluctuations in the patient’s condition, routinely assess the efficacy and safety of the treatment, and modify the insulin formulation or administration protocol as required. These measures are essential to mitigate the progression of complications and enhance the overall quality of life for patients [43].

## Limitations

The study has several limitations. Firstly, the FAERS database only includes AE reports submitted to the US FDA, which may be subject to biases such as underreporting and selective reporting. Secondly, the signal detection results merely indicate an association between the AE and the drug, not causality. Therefore, future studies should undertake in-depth case-control or cohort studies to incorporate important covariates, assess influencing factors, and evaluate the clinical outcomes of insulin-related AEs, as well as further validate the results of signal detection. Additionally, it is imperative to enhance basic research to elucidate the mechanisms and genetic foundations of insulin adverse reactions, providing a basis for precision medicine and individualized treatment strategies. Furthermore, it is necessary to enhance the development of pharmacovigilance systems, reinforcing the monitoring, identification, evaluation, and management of AEs to advance the scientific and effective handling of drug safety. Ultimately, only through multidisciplinary, multifaceted, and multicenter collaboration can the safety and efficacy of insulin therapy be continuously improved to better serve the vast majority of diabetes patients.

## Conclusion

This study provides comprehensive real-world evidence on the safety profile of IG, revealing specific risks in different populations. In the overall population, strong signals were detected for serious AEs related to blood glucose abnormalities, cutaneous amyloidosis, and hypoglycemic seizure, emphasizing the need for close monitoring and management of these potential complications. For pregnant women, hypoglycemia was the only strong signal, while medium and weak signals were identified for various congenital anomalies, pregnancy-related issues, and neonatal complications. The longer TTO of AEs in pregnant women suggests a need for extended monitoring throughout pregnancy. These findings highlight the importance of optimizing the benefit-risk profile of IG therapy across diverse patient populations. Further research is warranted to elucidate the underlying mechanisms and risk factors associated with IG-related AEs, especially in the context of pregnancy.

## Supporting information

S1 File This file contains S1-S6 Tables. **S1 Table**. The breakdown of the SMQ codes. S2 Table. The four algorithms used for signal detection. S3 Table. AE signals in various SOCs for IG used in the overall population. S4 Table. AE signals in various SOCs for IG used in pregnant women. S5 Table. TTO analysis of IG-related AEs at the PT level in the overall population. S6 Table. TTO analysis of IG-related AEs at the PT level in pregnant women.
(DOCX)
