# Supplementary material for: A disproportionality analysis of insulin glargine in the overall population and in pregnant women using the FDA adverse event reporting system (FAERS) database
Source: PLoS One. 2025 Sep 25;20(9):e0331489. doi: 10.1371/journal.pone.0331489 (PMC12463294; doi:10.1371/journal.pone.0331489)
Supplement: S1 File — S1 Table. The breakdown of the SMQ codes. S2 Table. The four algorithms used for signal detection. S3 Table. AE signals in various SOCs for IG used in the overall population. S4 Table. AE signals in various SOCs for IG used in pregnant women. S5 Table. TTO analysis of IG-related AEs at the PT level in the overall population. S6 Table. TTO analysis of IG-related AEs at the PT level in pregnant women. (DOCX) [file pone.0331489.s001.docx]

# **A disproportionality analysis of insulin glargine in the overall population and in pregnant women using the FDA adverse event reporting system (FAERS) database**

# Shaozhi Liu^1,*^, Jun Xu^1,*^, Zhongwen Yuan^1^, Zhengrong Mei^1^, Shengying Shi^1^, Jinjin Yin^1,#^, Yanhong Deng^1,#^

^1 Department of Pharmacy, Guangdong Provincial Key Laboratory of Major Obstetric Diseases, Guangdong Provincial Clinical Research Center for Obstetrics and Gynecology, The Third Affiliated Hospital, Guangzhou Medical University, Guangzhou, China.^

^* These authors contributed equally to this work.^

^#Corresponding author: Jinjin Yin: [yinjinjinsunny@163.com;](mailto:yinjinjinsunny@163.com;) Yanhong Deng: yhdeng309@sina.com.^

S1 Table . The breakdown of the SMQ codes.

| SMQ codes | SMQ name |
| --- | --- |
| 20000077 | congenital, familial, and genetic disorders |
| 20000186 | pregnancy, labor, and delivery complications, and risk factors |
| 20000190 | fetal disorders |
| 20000191 | neonatal disorders |
| 20000192 | termination of pregnancy and risk of abortion |
| 20000193 | normal pregnancy conditions and outcomes |

S2 Table. The four algorithms used for signal detection.

| **Algorithms** | **Equation** | **Criteria** |
| --- | --- | --- |
| ROR | ROR=ad/bc | lower limit of 95% CI>1, N≥3 |
|  | 95%CI=e^ln(ROR)±1.96(1/a+1/b+1/c+1/d)^0.5^ |  |
| PRR | PRR=a/(c+d)/c/(a+b) | PRR≥2, χ^2^≥4, N≥3 |
|  | χ^2^=[(ad-bc)^2](a+b+c+d)/[(a+b)(c+d)(a+c)(b+d)] |  |
| BCPNN | IC = log_2_a(a+b+c+d)/[(a+c)(a+b)] | IC025>0 |
|  | 95%CI= E(IC)+ 2V(IC)^0.5 |  |
| EBGM | EBGM=a(a+b+c+d)/(a+c)(a+b) | EBGM05>2 |
|  | 95%CI =e^ln(EBGM)±1.96(1/a+1/b+1/c+1/d)^0.5^ |  |

**Notes**: Equation: *a*, number of reports containing both the target drug and target adverse drug reaction; *b*, number of reports containing other

adverse drug reaction of the target drug; *c*, number of reports containing the target adverse drug reaction of other drugs; *d*, number of reports

containing other drugs and other adverse drug reactions.

**Abbreviations**: 95% CI, 95% confidence interval; N, the number of reports; *χ* 2 , chi-squared; IC, information component; IC025, the lower limit of 95% CI of the IC; *E*(*IC*), the *IC* expectations; *V*(*IC*), the variance of *IC*; *EBGM*, empirical Bayesian geometric mean; EBGM05, the lower limit of 95% CI of *EBGM*.

S3 Table. AE signals in various SOCs for IG used in the overall population.

| **System Organ Class(SOC)** | **Case reports** | **ROR(95% CI)** | **PRR(95% CI)** | **Chi_Square** | **IC(IC025)** | **EBGM(EBGM05)** |
| --- | --- | --- | --- | --- | --- | --- |
| Product issues | 13238 | 3.80(3.73,3.87) | 3.64(3.58,3.70) | 25347.8 | 1.85(1.82) | 3.60(3.54) |
| Eye disorders | 14410 | 3.34(3.28,3.40) | 3.19(3.14,3.24) | 21842.9 | 1.66(1.64) | 3.16(3.11) |
| Investigations | 33861 | 2.65(2.62,2.68) | 2.40(2.38,2.43) | 29311.5 | 1.26(1.24) | 2.39(2.36) |
| Metabolism and nutrition disorders | 11391 | 2.36(2.32,2.40) | 2.29(2.25,2.33) | 8399.76 | 1.19(1.16) | 2.28(2.24) |
| Injury, poisoning and procedural complications | 48258 | 2.34(2.32,2.36) | 2.06(2.04,2.08) | 29018 | 1.04(1.02) | 2.05(2.03) |
| Ear and labyrinth disorders | 1942 | 1.96(1.87,2.05) | 1.95(1.86,2.04) | 892.24 | 0.96(0.89) | 1.94(1.85) |
| Surgical and medical procedures | 4931 | 1.62(1.58,1.67) | 1.61(1.57,1.66) | 1146.57 | 0.68(0.64) | 1.61(1.56) |
| Social circumstances | 1360 | 1.28(1.21,1.35) | 1.28(1.21,1.35) | 81.32 | 0.35(0.27) | 1.27(1.21) |
| Nervous system disorders | 17500 | 0.88(0.87,0.90) | 0.89(0.88,0.90) | 250.69 | -0.16(-0.19) | 0.89(0.88) |
| Cardiac disorders | 4827 | 0.79(0.76,0.81) | 0.79(0.77,0.81) | 276.63 | -0.34(-0.38) | 0.79(0.77) |
| General disorders and administration site conditions | 30145 | 0.71(0.70,0.72) | 0.75(0.74,0.76) | 3011.85 | -0.41(-0.43) | 0.75(0.74) |
| Renal and urinary disorders | 3048 | 0.69(0.66,0.71) | 0.69(0.67,0.72) | 429.62 | -0.53(-0.58) | 0.69(0.67) |
| Pregnancy, puerperium and perinatal conditions | 526 | 0.53(0.48,0.57) | 0.53(0.48,0.57) | 223.21 | -0.92(-1.04) | 0.53(0.49) |
| Infections and infestations | 6335 | 0.51(0.50,0.53) | 0.53(0.51,0.54) | 2850.01 | -0.92(-0.96) | 0.53(0.51) |
| Musculoskeletal and connective tissue disorders | 6225 | 0.51(0.50,0.52) | 0.52(0.51,0.53) | 2881.59 | -0.94(-0.97) | 0.52(0.51) |
| Endocrine disorders | 298 | 0.51(0.46,0.57) | 0.51(0.46,0.57) | 139.51 | -0.97(-1.13) | 0.51(0.46) |
| Vascular disorders | 2484 | 0.50(0.48,0.52) | 0.50(0.48,0.52) | 1243.96 | -0.99(-1.05) | 0.50(0.48) |
| Respiratory, thoracic and mediastinal disorders | 5221 | 0.47(0.46,0.48) | 0.48(0.47,0.49) | 3054.78 | -1.05(-1.09) | 0.48(0.47) |
| Neoplasms benign, malignant and unspecified (incl cysts and polyps) | 2876 | 0.46(0.45,0.48) | 0.47(0.45,0.49) | 1759.02 | -1.09(-1.14) | 0.47(0.45) |
| Skin and subcutaneous tissue disorders | 5758 | 0.45(0.44,0.46) | 0.46(0.45,0.48) | 3751.82 | -1.10(-1.14) | 0.47(0.45) |
| Hepatobiliary disorders | 917 | 0.43(0.40,0.46) | 0.43(0.41,0.46) | 681.73 | -1.20(-1.30) | 0.44(0.41) |
| Psychiatric disorders | 5366 | 0.40(0.39,0.41) | 0.41(0.40,0.42) | 4827.64 | -1.28(-1.32) | 0.41(0.40) |
| Immune system disorders | 972 | 0.38(0.36,0.41) | 0.38(0.36,0.41) | 968.97 | -1.38(-1.47) | 0.39(0.36) |
| Congenital, familial and genetic disorders | 247 | 0.35(0.31,0.40) | 0.35(0.31,0.40) | 294.41 | -1.50(-1.68) | 0.35(0.31) |
| Gastrointestinal disorders | 6997 | 0.34(0.33,0.34) | 0.36(0.35,0.36) | 8867.21 | -1.48(-1.52) | 0.36(0.35) |
| Reproductive system and breast disorders | 366 | 0.18(0.16,0.19) | 0.18(0.16,0.20) | 1417.73 | -2.50(-2.64) | 0.18(0.16) |
| Blood and lymphatic system disorders | 457 | 0.12(0.11,0.13) | 0.12(0.11,0.13) | 3071.4 | -3.08(-3.21) | 0.12(0.11) |

Note: ranked by case reports

S4 Table. AE signals in various SOCs for IG used in pregnant women.

| **System Organ Class(SOC)** | **Case reports** | **ROR(95% CI)** | **PRR(95% CI)** | **Chi_Square** | **IC(IC025)** | **EBGM(EBGM05)** |
| --- | --- | --- | --- | --- | --- | --- |
| Metabolism and nutrition disorders | 221 | 6.80(5.92,7.81) | 6.33(5.58,7.19) | 992.34 | 2.65(2.41) | 6.26(5.45) |
| Injury, poisoning and procedural complications | 1001 | 1.79(1.66,1.94) | 1.50(1.43,1.58) | 222.5 | 0.59(0.48) | 1.50(1.39) |
| Endocrine disorders | 8 | 1.75(0.87,3.50) | 1.74(0.87,3.49) | 2.53 | 0.80(-0.28) | 1.74(0.87) |
| Surgical and medical procedures | 85 | 1.60(1.29,1.98) | 1.58(1.28,1.95) | 18.34 | 0.66(0.33) | 1.58(1.27) |
| Product issues | 18 | 1.45(0.91,2.30) | 1.45(0.91,2.29) | 2.47 | 0.53(-0.17) | 1.44(0.91) |
| Investigations | 146 | 1.43(1.21,1.70) | 1.41(1.21,1.65) | 18.17 | 0.50(0.25) | 1.41(1.19) |
| Eye disorders | 28 | 1.42(0.98,2.06) | 1.41(0.98,2.04) | 3.38 | 0.50(-0.06) | 1.41(0.97) |
| Hepatobiliary disorders | 22 | 1.32(0.87,2.01) | 1.32(0.87,2.00) | 1.67 | 0.39(-0.23) | 1.31(0.86) |
| Pregnancy, puerperium and perinatal conditions | 507 | 1.29(1.17,1.42) | 1.23(1.14,1.33) | 26.14 | 0.30(0.16) | 1.23(1.12) |
| Vascular disorders | 33 | 1.06(0.75,1.49) | 1.06(0.75,1.49) | 0.11 | 0.08(-0.42) | 1.06(0.75) |
| Respiratory, thoracic and mediastinal disorders | 81 | 0.94(0.75,1.17) | 0.94(0.76,1.17) | 0.28 | -0.08(-0.41) | 0.94(0.76) |
| Renal and urinary disorders | 18 | 0.90(0.56,1.43) | 0.90(0.57,1.42) | 0.21 | -0.16(-0.81) | 0.90(0.56) |
| Congenital, familial and genetic disorders | 175 | 0.76(0.65,0.89) | 0.78(0.67,0.90) | 12.19 | -0.36(-0.59) | 0.78(0.67) |
| Reproductive system and breast disorders | 27 | 0.76(0.52,1.11) | 0.76(0.52,1.11) | 2.05 | -0.39(-0.93) | 0.76(0.52) |
| Infections and infestations | 52 | 0.62(0.47,0.81) | 0.63(0.48,0.82) | 11.97 | -0.67(-1.07) | 0.63(0.48) |
| Cardiac disorders | 31 | 0.58(0.41,0.82) | 0.58(0.41,0.83) | 9.47 | -0.78(-1.27) | 0.58(0.41) |
| Gastrointestinal disorders | 62 | 0.50(0.39,0.64) | 0.51(0.40,0.65) | 30.3 | -0.97(-1.32) | 0.51(0.40) |
| Blood and lymphatic system disorders | 11 | 0.49(0.27,0.89) | 0.49(0.27,0.89) | 5.77 | -1.02(-1.79) | 0.49(0.27) |
| Nervous system disorders | 54 | 0.44(0.33,0.57) | 0.45(0.34,0.58) | 38.62 | -1.16(-1.54) | 0.45(0.34) |
| General disorders and administration site conditions | 115 | 0.39(0.33,0.47) | 0.42(0.35,0.50) | 103.87 | -1.26(-1.53) | 0.42(0.35) |
| Neoplasms benign, malignant and unspecified (incl cysts and polyps) | 3 | 0.27(0.09,0.83) | 0.27(0.09,0.83) | 5.97 | -1.89(-3.04) | 0.27(0.09) |
| Ear and labyrinth disorders | 2 | 0.24(0.06,0.95) | 0.24(0.06,0.96) | 4.86 | -2.06(-3.31) | 0.24(0.06) |
| Skin and subcutaneous tissue disorders | 15 | 0.23(0.14,0.38) | 0.23(0.14,0.39) | 38.45 | -2.09(-2.75) | 0.23(0.14) |
| Immune system disorders | 3 | 0.19(0.06,0.58) | 0.19(0.06,0.58) | 10.7 | -2.42(-3.54) | 0.19(0.06) |
| Social circumstances | 2 | 0.15(0.04,0.62) | 0.15(0.04,0.62) | 9.31 | -2.69(-3.88) | 0.15(0.04) |
| Musculoskeletal and connective tissue disorders | 15 | 0.13(0.08,0.21) | 0.13(0.08,0.22) | 87.59 | -2.90(-3.54) | 0.13(0.08) |
| Psychiatric disorders | 13 | 0.10(0.06,0.17) | 0.10(0.06,0.18) | 106.91 | -3.28(-3.96) | 0.10(0.06) |

Note: ranked by case reports.

S5 Table. TTO analysis of IG-related AEs at the PT level in the overall population.

| **SOC/PT** | **Case reports** | **Median (IQR)** |
| --- | --- | --- |
| **Metabolism and nutrition disorders** |  |  |
| Hypoglycaemia | 3689 | 131(2,628) |
| Ketoacidosis | 266 | 335.5(31,1088) |
| Insulin resistance | 86 | 2(0,159) |
| Shock hypoglycaemic | 49 | 77.5(0,731) |
| Hypoglycaemia neonatal | 40 | 200.5(27,986) |
| Hypoglycaemia unawareness | 34 | 200.5(27,986) |
| Ketosis | 30 | 660.5(124.5,1875.5) |
| Dawn phenomenon | 17 | 51(0,61) |
| Hyperinsulinaemic hypoglycaemia | 16 | 0(0,0) |
| **Investigations** |  |  |
| Blood glucose decreased | 4213 | 22(1,583) |
| Blood glucose abnormal | 2165 | 15(1,161) |
| Blood glucose fluctuation | 1188 | 158(5,731) |
| Glycosylated haemoglobin decreased | 88 | 135.5(0,211) |
| Anti-insulin antibody positive | 30 | 63(15,984) |
| Blood ketone body | 26 | 83(1,912) |
| Blood ketone body increased | 26 | 695(172,1218) |
| Blood insulin increased | 20 | 200(90,310) |
| Blood ketone body present | 10 |  |
| **General disorders and administration site conditions** |  |  |
| Injection site pain | 3496 | 15(0,390) |
| Injection site haemorrhage | 1236 | 28(0,365) |
| Injection site bruising | 976 | 1(0,138.5) |
| Injection site discolouration | 179 | 14(8,365) |
| Hunger | 154 | 14.5(0.5,366.5) |
| Injection site injury | 150 | 0(0,548) |
| Injection site scar | 59 | 577(577,577) |
| Injection site atrophy | 43 | 16(3,1217) |
| Injection site hypertrophy | 22 | 16(0,364) |
| **Surgical and medical procedures** |  |  |
| Cardiac operation | 249 | 72(0,486) |
| Cataract operation | 244 | 1886(1886,1886) |
| Eye operation | 152 | 389.5(151,1585) |
| Coronary artery bypass | 107 | 501.5(222,924) |
| Vascular graft | 99 | 304(22,1308) |
| Eye laser surgery | 28 | 699(122,730) |
| Retinal operation | 11 | 414.5(130,699) |
| Laser therapy | 11 | 297(229,365) |
| **Eye disorders** |  |  |
| Visual impairment | 5698 | 236(1,1004) |
| Cataract | 1525 | 366(92,1004) |
| Blindness | 1004 | 365(1,1249) |
| Visual acuity reduced | 809 | 365(10,1096) |
| Eye disorder | 740 | 365.5(0,882) |
| Glaucoma | 390 | 402(66,1461) |
| Eye haemorrhage | 380 | 365(31,924) |
| Macular degeneration | 369 | 792(153,1826) |
| Blindness unilateral | 323 | 365(12,1096) |
| Retinopathy | 182 | 120(0,1096) |
| Retinal haemorrhage | 115 | 365(103,670) |
| Retinal disorder | 69 | 61(0,2557) |
| Retinopathy proliferative | 10 | 185(105,265) |
| **Social circumstances** |  |  |
| Disability | 533 | 731(0,5051) |
| Wheelchair user | 120 | 1789(365.5,2900) |
| Corrective lens user | 45 | 348.5(1,696) |
| **Nervous system disorders** |  |  |
| Cerebrovascular accident | 2003 | 361.5(27.5,841) |
| Dementia | 439 | 631(92,1842) |
| Hypoglycaemic coma | 233 | 70.5(1,372.5) |
| Hypoglycaemic unconsciousness | 208 | 126.5(20,659.5) |
| Dementia Alzheimer's type | 188 | 882(316,1108.5) |
| Hypoglycaemic seizure | 117 | 123(29,598) |
| Hypoglycaemic encephalopathy | 26 | 172.5(72,663) |
| **Skin and subcutaneous tissue disorders** |  |  |
| Lipodystrophy acquired | 38 | 1565(14,3384.5) |
| Lipohypertrophy | 31 | 0(0,4.5) |
| Cutaneous amyloidosis | 12 | 820.5(37,1604) |
| **Injury, poisoning and procedural complications** |  |  |
| Exposure via skin contact | 169 | 0(0,0) |
| Lack of injection site rotation | 49 | 8(1,1840.5) |
| **Other SOC** |  |  |
| Hypoacusis | 1393 | 335.5(1,1461) |
| Frustration tolerance decreased | 151 | 14(0,19) |
| Pancreatic disorder | 134 | 172(17,1096) |

Note: interquartile range.

S6 Table. TTO analysis of IG-related AEs at the PT level in pregnant women.

| **System Organ Class(SOC)/Preferred Term(PT)** | **Case reports** | **Median (IQR)** |
| --- | --- | --- |
| **Metabolism and nutrition disorders** | | |
| Hypoglycaemia | 73 | 1128(118,2025) |
| Hypoglycaemia neonatal | 40 | 220(0,270) |
| Decreased insulin requirement | 3 | 2025(2025,2025) |
| Underweight | 3 | 132(132,132) |
| **Nervous system disorders** | | |
| Hypoglycaemic seizure | 4 | 30.5(0,61) |
| **Investigations** | | |
| Blood glucose decreased | 13 | 0(0,0) |
| Blood glucose fluctuation | 9 | 245(0,326) |
| **Injury, poisoning and procedural complications** | | |
| Foetal exposure during pregnancy | 518 | 47(0,252) |
| **Congenital, familial and genetic disorders** | | |
| Kidney malformation | 5 | 26(0,26) |
| Congenital bladder anomaly | 3 | 0(0,0) |
| Gastrointestinal malformation | 3 | 558(558,558) |
| **Pregnancy, puerperium and perinatal conditions** | | |
| Abortion | 18 | 41(24,47) |
| Jaundice neonatal | 16 | 112(17,228) |
| Foetal distress syndrome | 16 | 193(186,193) |
| Foetal disorder | 8 | 502(382,558) |
| Polyhydramnios | 8 | 648(169,1651) |
| Foetal hypokinesia | 7 | 2025(2025,2025) |
| Abnormal labour | 3 | 41(24,47) |
| **Infections and infestations** | | |
| Kidney infection | 3 | 1327(1327,1327) |
| **General disorders and administration site conditions** | | |
| Injection site pain | 17 | 0(0,0.5) |
| Injection site haemorrhage | 3 | 0(0,0) |
| **Respiratory, thoracic and mediastinal disorders** | | |
| Respiratory arrest | 3 | 186(186,186) |
| **Hepatobiliary disorders** | | |
| Jaundice | 10 | 1087(149,2025) |

Note: interquartile range.
